# Sensitivity, specificity and predictive values of hearing loss to different audiometric mean values

**DOI:** 10.1016/S1808-8694(15)30539-5

**Published:** 2015-10-19

**Authors:** Karin Christine de Freitas Kasper Calviti, Liliane Desgualdo Pereira

**Affiliations:** 1 MSc; Speech and Hearing Therapist - Federal University of São Paulo; 1 Associate Professor of Hearing Disorders- Federal University of São Paulo - UNIFESP. Federal University of São Paulo - UNIFESP

**Keywords:** audiometry, elderly, presbycusis

## Abstract

Hearing loss in the elderly is one of the most incapacitating communication disorders, preventing them to fully perform their tasks in society.

**Aim:**

This study aimed to determine what is the best tool that together with the audiometric tests better represents the hearing loss reported by the elderly and which frequencies in the audiometric test must be considered to determine the hearing loss degree.

**Study:**

Clinical prospective.

**Materials and Methods:**

71 elderly with ages between 60 and 82 years old were assessed. The subjects were submitted to a conventional audiometric assessment and a Hearing Handicap Inventory for the Elderly (HHIE) questionnaire. Three audiometric averages were analyzed and compared with the results obtained in the complete form (HHIE) and in its reduced form (HHIE-S).

**Results:**

specificity showed values between 43.5% and 58.5% for HHIE with the different audiometric averages and values between 50% and 63.4% for the HHIE-S.

**Conclusion:**

audiometric average pure tone thresholds in the frequencies of 4kHz and 6kHz found in the audiometric assessment did not contribute to the self-reported hearing handicap perception. The correlation between HHIE-S and PTA1 had the best specificity (63.4%) and best positive predictive value (62.5%).

## INTRODUCTION

Humans are living longer thanks to mankind social, economic and scientific developments. Although a desire of most people, it may result in living with disabilities and dependence. The epidemiological transition resulting from the demographic change has altered the morbi-mortality profile of the populations which went through the population aging process (increase in the ratio of elderly citizens). Possible results are the increase in the prevalence of chronic-degenerative disorders, with its sequelae and complications, causing disability, dependence, the need of care for longer periods even in specialized institutions, and many others such as social support, social networks, loss of social roles, loneliness, solitude, depression, loss of autonomy and of personal meaning, and the lack of meaning for life itself. One major challenge that increased longevity brings us is that of furthering survival with an increasingly better quality of life.^1^

Hearing loss causes one of the most disabling communication disorders in the elderly, preventing them from fully performing in society. Of all the sensorial deficits faced by the elderly, communication impairment caused by hearing loss can be one of the most frustrating. It is common to see hearing decline followed by a frustrating speech understanding in the elderly. Senior citizens with hearing impairment have a reduction in auditory sensitivity and a reduction in speech intelligibility at supra-threshold levels, which seriously compromises their verbal communication processes. Since hearing loss starts gradually and does not manifest as a disease, especially in its initial stages, it is not perceived by the patient. It is common for individuals to state they can hear, but can not understand well what is being said. Elderly individuals have a greater difficulty with speech under noise and reverberation; and they also have greater difficulty of complementing their hearing with visual information obtained by means of lip reading. This persistent failure in understanding speech can result in frustration and disheartening, which can lead to resignation.^1^

The gold standard used to assess hearing is tonal audiometry, a method which does not analyses qualitatively the hearing loss, and it only classifies the level of hearing loss in the different frequencies tested.

In 1982, Ventry and Weinstein^2^ introduced a questionnaire for psychosocial self-perception of the hearing loss in the elderly as a complement to tonal audiometry in the hearing aid fitting process efficacy. This questionnaire, the Hearing Handicap Inventory for the Elderly (HHIE) was made up of 25 items broken down into social and emotional aspects. In 1983, the authors published an abridged version of the HHIE, the Hearing Handicap Inventory for the Elderly - Screening (HHIE-S) with 10 questions, also broken down into social and emotional aspects, proposed as a screening used to detect the level of complaint by the elderly.^3^

Studies have attempt to analyze and validate instruments that could evaluate to the hearing loss in the elderly and to measure the hearing complaint.^4^, ^5^

Corrêa, Russo ^6^, Sindhusake et al.^7^, Gates et al.^8^, Pizan, Iório^9^, Yueh et al.^10^ compared the handicap degree with the auditory sensitivity and observed the correlation between the measures.

Both procedures have been broadly utilized and the HHIE-S is the one most employed because it is more practical; however, it is not well established if the HHIE, even being longer, would not be a more reliable tool to assess the hearing complaint.

The proposal of this study was to find out the best instrument which together with the audiometry better represents the elderly patient's complaint, and which tonal audiometry frequencies must be considered in order to assign the degree of hearing loss.

## MATERIALS AND METHODS

This paper was referred to the Ethics in Research Committee for appreciation and was approved under protocol # 0823/06 on June 23, 2006.

We evaluated 71 elderly patients, aged between 60 and 82 years, 48 women with mean age of 71.16 years and 23 men, with mean age of 73.91. This study started after approval by the head of the department of this institution where these elderly were assessed.

These senior citizens were invited to participate by means of an invitation letter and by agreeing they signed an Informed Consent Form; they came referred to the audiology service by their physician requesting audiologic evaluation and were then invited to undergo the other tests proposed in the study - which were all done in the same day.

We used inclusion and exclusion criteria to select the individuals participating in this study. Inclusion criteria were: elderly with ages equal to or above 60 years; elderly individuals with normal hearing and some with mild to moderate sensorineural hearing loss. The exclusion criteria were: severe to profound hearing loss and air conduction hearing loss.

All the individuals were submitted to basic audiologic evaluation and the HHIE questionnaire.

In the basic audiologic evaluation we performed the following procedures: threshold tonal audiometry; SRT - Speech Reception Threshold - attained by means of trisyllable words^11^, speech recognition percentage index (SRPI) - obtained by using monosyllabic words^11^ and immittance studies.

The hearing thresholds considered normal were those found up to 25 dB HL (ISO 7566 Standard) in all the sound frequencies assessed, 250, 500, 1000, 2000, 3000, 4000, 6000 and 8000 Hz, based on Silman, Silverman.^12^

In order to classify the degree of hearing loss, we adopted the recommendation by Davis^13^, based on the mean values of the tonal thresholds obtained from the sound frequencies of 500, 1000 and 2000 Hz. Nevertheless, in order to check the importance of the 4kHz and 6kHz thresholds, this classification was employed for the tonal threshold mean values obtained in the sound frequencies of 500, 1000 and 2000 Hz (PTA 1), for the mean values of the tonal thresholds obtained in the sound frequencies of 500, 1000, 2000 and 4000 Hz (PTA 2) and for the mean values of the tonal thresholds obtained in the sound frequencies of 500, 1000, 2000, 4000 and 6000 Hz (PTA 3).

Thus, for this study, auditory sensitivity was classified and identified for statistical analysis purposes, as “normal hearing” for hearing thresholds of up to 25 dB HL and as “altered hearing” for the mean hearing thresholds above 25 dB HL.

The individuals selected for this study were also submitted to the Hearing Handicap Inventory for the Elderly - HHIE questionnaire, used in the format of an interview, in order to list the social and non-auditory aspects resulting from the handicap and hearing loss, which limits or prevents the individual from properly performing his/her daily activities and which compromise his/her family, work and social lives, as observed by Russo.^14^

This auditory handicap questionnaire proposed by Ventry, Weinstein^2^ and adapted to Brazilian Portuguese by Wieselberg^15^ is made up of 25 questions which must be answered by checking one alternative among “yes”, “no” and “sometimes”.

In order to establish the handicap level we followed the evaluation and scoring criteria proposed by Ventry, Weinstein2: YES = 4 points (%); NO = 0 points (%); SOMETIMES = 2 points (%).

Through the answers obtained in this questionnaire, the individuals were distributed in three groups. The criteria followed for this classification was the score obtained in the questionnaire, which follows the classification as shown on Chart 1.

**Chart 1:** Handicap perception classification.

Handicap perception classification	Handicap index (%)	Identification in the results
There is no handicap perception	From 0 % to 16 %	Without perception
Mild/moderate handicap perception	From 18 % to 42 %	With perception
Severe/significant handicap perception	Above 42 %	

After employing the complete questionnaire we extracted the 10 items corresponding to the HHIE-S for analysis, and we obtained the results for each individual regarding the HHIE and HHIE-S.

Besides this quantitative classification, the questionnaire was broken down into emotional (E) and social (S) aspects, which were considered for the qualitative analysis in both the procedures, the HHIE and the HHIE-S.

In order to realize the elected procedures, we used the following material: 1) Amplaid A321 audiometer with a TDH39 phone and Mx41/AR pad, calibrated according to the EM ISO 389 (1995), ANSI S3.6 91996 and ISO 389-3 (1994) standards. 2) Impedance meter from Interacoustics, model AT 235/425 calibrated according to the ANSI S3.6 – 1996/ISSO 8253-1. 3 norm) and the Hearing Handicap Inventory for the Elderly questionnaire.

For sample analysis we used the Anderson-Daling normality test, showed on histogram. We used the chi-squared non-parametric test for independence and in analysis complementation we calculated the sensitivity and specificity statistical values and the predictive value of the elderly hearing complaint in relation to the different audiometric differences (PTA1, PTA2 and PTA3).

Sensitivity was defined as the percentage of patients complaining of hearing loss among those in whom the audiometric exam showed hearing loss. The specificity was the percentage of patients without hearing complaint among those with normal hearing.

The positive and negative predictive values were defined, respectively, as the likelihood of the patient having hearing loss among those with hearing complaints and of presenting normal audiometric values among those without hearing complaints.

## RESULTS

The results are shown on 5 Tables, according to the text that follows.

On Table 1 we see the correlations between the auditory thresholds, SRT, SRPE and different audiometric mean values obtained by means of audiometric evaluations and the patient's age with his/her hearing compliant which was obtained by means of applying The Hearing Handicap Inventory for the Elderly - HHIE, in its complete and reduced form.

**Table 1:** Correlation between the quantitative aspect of the HHIE and HHIE-S questionnaires with audiologic aspects and age.

AUDIOMETRY/AGE	HHIE		HHIE-S	
	Correlation (%)	p-value	Correlation (%)	p-value
250 Hz	50,8%	<0,001*	53,9%	<0,001*
500 Hz	56,1%	<0,001*	59,1%	<0,001*
1 kHz	54,9%	<0,001*	58,8%	<0,001*
2 kHz	45,3%	<0,001*	48,8%	<0,001*
4 kHz	39,9%	<0,001*	41,5%	<0,001*
6 kHz	37,2%	<0,001*	41,2%	<0,001*
8 kHz	28,0%	<0,001*	30,8%	<0,001*
SRT	54,4%	<0,001*	58,6%	<0,001*
IPRF	-36,3%	<0,001*	-35,9%	<0,001*
PTA 1	48,2%	<0,001*	51,4%	<0,001*
PTA 2	62,1%	<0,001*	63,2%	<0,001*
PTA 3	51,7%	<0,001*	57,5%	<0,001*
Age	17,7%	0,035*	16,4%	0,051#

Correlation test.

Legend: * statistically significant.

# Tendency towards significance

Table 2 shows the correlation between the HHIE and the audiometric mean values at 500, 1000 e 2000 Hz (PTA 1), at 500, 1000, 2000 and 4000 Hz (PTA 2) and at 500, 1000, 2000, 4000 and 6000 Hz (PTA 3).

**Table 2:** Correlation between the HHIE and the different audiometric mean values.

HHIE		Normal		Altered		Total		p-value
		N	%	N	%	N	%	
	Without perception	27	90,0%	17	41,5%	44	62,0%	
PTA 1	With perception	3	10,0%	24	58,5%	27	38,0%	<0,001*
	Total	30	42,3%	41	57,7%	71	100%	
	Without perception	13	100%	31	53,4%	44	62,0%	
PTA 2	With perception	0	0,0%	27	46,6%	27	38,0%	0,002*
	Total	13	18,3%	58	81,7%	71	100%	
	Without perception	9	100%	35	56,5%	44	62,0%	
PTA 3	With perception	0	0,0%	27	43,5%	27	38,0%	0,012*
	Total	9	12,7%	62	87,3%	71	100%	

Chi squared test.

Legend: * statistically significant.

Table 3 shows the sensitivity, specificity and positive and negative predictive values for each one audiometric mean value by means of the HHIE.

**Table 3:** Sensitivity, specificity and positive and negative predictive values for each audiometric mean value with the use of the HHIE.

HHIE	PTA 1	PTA 2	PTA 3
Accuracy	71.8%	56.3%	50.7%
Sensitivity	90.0%	100%	100%
Specificity	58.5%	46.6%	43.5%
+ Predictive value	61.4%	29.5%	20.5%
- Predictive value	88.9%	100%	100%

Table 4 shows the correlation between the HHIE-S and the audiometric mean values at 500, 1000 and 2000 Hz (PTA 1), at 500, 1000, 2000 and 4000 Hz (PTA 2) and at 500, 1000, 2000, 4000 e 6000 Hz (PTA 3).

**Table 4:** Correlation between the HHIE-S and the different audiometric mean values.

HHIE-S		Normal		Altered		Total		p-value
	N	%		N	%	N	%	
	W/out perception	25	83,3%	15	36,6%	40	56,3%	
PTA 1	With perception	5	16,7%	26	63,4%	31	43,7%	<0,001*
	Total	30	42,3%	41	57,7%	71	100%	
	W/out perception	13	100%	27	46,6%	40	56,3%	
PTA 2	With perception	0	0,0%	31	53,4%	31	43,7%	<0,001*
	Total	13	18,3%	58	81,7%	71	100%	
	W/out perception	9	100%	31	50,0%	40	56,3%	
PTA 3	With perception	0	0,0%	31	50,0%	31	43,7%	0,005*
	Total	9	12,7%	62	87,3%	71	100%	

Chi squared test

Legend: * statistically significant.

Table 5 shows the sensitivity, specificity and positive and negative predictive values for each audiometric mean values with the HHIE-S.

**Table 5:** Sensitivity, specificity and positive and negative predictive values for each audiometric mean value with the HHIE-S.

HHIE-S	PTA 1	PTA 2	PTA 3
Accuracy	71,8%	62,0%	56,3%
Sensitivity	83,3%	100%	100%
Specificity	63,4%	53,4%	50,0%
+ Predictive value	62,5%	32,5%	22,5%
- Predictive value	83,9%	100%	100%

## DISCUSSION

The present study correlated the auditory thresholds, SRT, SRPI and different audiometric mean values obtained by means of an audiometric evaluation and age with the elderly hearing complaint by means of the Hearing Handicap Inventory for the Elderly - HHIE, both the complete and the abridged versions.

In the present investigation both the HHIE-S score and the HHIE score presented a correlation with the auditory thresholds. There was a statistically significant correlation regarding all the auditory thresholds for the SRT and the SRPI (Table 1). Both the HHIE and the HHIE-S showed good sensitivity and medium specificity. Sensitivity was of 90%, 100% and 100% regarding the use of HHIE with the different audiometric mean values, respectively PTA1, PTA 2 and PTA 3; and 83.3%, 100% and 100% regarding the use of HHIE-S also with the different audiometric mean values, respectively: PTA1, PTA 2 and PTA 3. Specificity presented values of 43.5% and 58.5% in order to use the HHIE depending on the audiometric mean values and 50% and 63.4% in order to use the HHIE-S (Table 2, Table 3, Table 4, Table 5). There was a tendency for age to present a statistically significant correlation with the auditory complaint assessment, suggesting likelihood that the higher the age, the greater the auditory complaint (Table 1), and this was also noticed by Wiley et al.^16^ who assessed 3,178 adults with ages between 48 and 92 years using auditory thresholds, speech recognition and HHIE-S. Wiley et al.^16^ also reported a greater HHIE-S score proportional to the hearing loss.

Pinzan-Faria, Iório^9^ who investigated the correlation between auditory sensitivity and the handicap level perceived by the 112 elderly with age starting at 65 years also noticed significance between the handicap level and the auditory sensitiveness.

Gates et al.^8^ compared 2 screening methods to assess the hearing loss impairment in the elderly. They assessed 546 senior citizens submitted to biannual audiometry and the two screening methods - which were the 10 questions from the Hearing Handicap Inventory for the Elderly-Screening (HHIE-S) and one global question: “Do you have any hearing problem at the moment?” The gold standard was the audiogram showing the 40dBHL or higher pure tone threshold value, or in 2 kHz in one ear or 1 or 2 kHz in both ears. Both methods were compared with the gold standard in terms of specificity, sensitivity and predictive values.

Gates et al.8 reported that the 10 HHIE-S items presented 35% sensitivity and 94% specificity; differently from what we observed in this present study, in which sensitivity varied between 90% and 100%, depending on the audiometric mean value adopted. The global subjective value presented a higher sensitivity (71%); however, lower specificity (71%) than the HHIE-S, the authors concluded that the global value of the hearing loss was more effective than the detailed questionnaire to identify elderly individuals with unknown auditory handicap.

For the two auditory complaint assessments (HHIE and HHIE-S) with the different audiometric mean values we found the positive and negative predictive values (Table 3, Table 5) which suggest a greater likelihood (62.5%) of the patient having a hearing complaint and have hearing loss when using the HHIE-S to assess the hearing complaint and the hearing loss in the 500Hz, 1,000Hz and 2,000Hz thresholds.

The audiometric mean values involving higher frequencies such as 4kHz and 6kHz, PTA 2 and PTA 3 respectively, have a 100% negative predictive value for the two ways used to assess the auditory complaint, that is, none of the elderly without hearing loss for these audiometric mean values (PTA > 25dB) will have an auditory complaint. This also suggests that the 4kHz and/or 6Khz thresholds can raise the audiometric mean without relevant impact on the 500Hz, 1kHz and 2kHz frequencies, because when the mean value of these thresholds are higher than 25dB there is a 62.5% likelihood of the patient complaining of hearing loss, compared to 32.5% for the PTA 2 and 22.5% for the PTA 3.

Sindhusake et al.^7^, who also did a similar study, compared the use of a simple question and the HHIE-S questionnaire in order to identify individuals with hearing loss with the pure tone audiometry gold standard. The authors evaluated 2,015 individuals between 55 and 99 years who were part of a study group (The Blue Mountains Hearing Study) held between 1997 and 1999. All the patients answered the question: “Do you think you have hearing loss?” answered the HHIE-S and did the audiometry test. The question employed and the HHIE-S questionnaire were compared with the mean value of frequencies 500; 1,000; 2,000 and 4,000 kHz (PTA). The authors reported that the HHIE-S with score below eight had little sensitivity, but high specificity and positive predictive values.

In the present study we observed a high sensitivity, medium specificity and low positive predictive value (32.5%) when the HHIE-S questionnaire was compared to the mean values of frequencies 500; 1,000; 2,000 and 4,000 kHz (PTA 2 in the present study); this does not depend on the score. Regarding the other audiometric mean values and the use of the entire HHIE, we observed high sensitivity, despite the low positive predictive value when correlated with PTA 1 and PTA2. Nevertheless, Sindhusake et al.^7^ concluded that the HHIE-S presented enough sensitivity and specificity to assess the hearing loss prevalence.

Pinzan-Faria, Iório^9^, who investigated the correlation between the auditory sensitivity and the self-perceived handicap level, and did tonal threshold audiometry to study auditory thresholds in the frequencies of 250 through 8,000 Hz, speech recognition threshold (SRT), speech recognition percentage index (SRPI) with monosyllable words, employed the HHIE-S questionnaire in order to establish the handicap level, and also, as we did here, they classified the hearing loss according to Davis^13^, who used a simple average of the 500; 1,000 and 2,000 Hz frequencies from the best ear. The authors observed that there is an 84% likelihood of hearing loss in elderly patients with significant handicap perception.

Lichtennstein et al.^4^, who compared the performance of 178 senior citizens older than 65 years in the Hearing Handicap Inventory for the Elderly - Screening Version (HHIE-S) with 5 different hearing loss criteria commonly used in the clinic in order to check HHIE-S validity as a test to identify hearing loss in the elderly, noticed that in the higher HHIE-S score range there is a higher likelihood of the subject presenting a hearing loss matching the criterion utilized, and they concluded that the HHIE-S is a valid tool to identify hearing loss in the elderly, with a lower sensitivity than the other instruments used.

Similarly to the other studies mentioned, the present one studied the correlation between audiometric thresholds with the HHIE-S. Moreover, the present study investigated the correlation between the audiometric thresholds with the HHIE compared to the HHIE-S and its sensitivities and specificities for the three different audiometric mean values from: 500Hz, 1kHz and 2kHz; mean values from 500Hz, 1kHz, 2kHz and 4kHz and mean values from 500Hz, 1kHz, 2kHz, 4kHz and 6kHz.

## CONCLUSION

We could then conclude that the inclusion of the 4Khz and 6kHz in the audiometric mean values for hearing assessment purposes did not contribute to the perception of auditory complaints and that both the HHIE and the HHIE-S are good tools to assess the hearing complaint, and the correlation between HHIE-S and PTA1 have the better specificity (63.4%) and the best positive predictive value (62.5%).

## References

[1] Paschoal SMP, Jacob Filho W, Amaral JRG (2005). Avaliação Global do Idoso: Manual da Liga da GAMIA.

[2] Ventry IM, Weinstein BE (1982). The Hearing Handicap Inventory for the Elderly: a New Tool. Ear Hear..

[3] Ventry I, Weinstein B (1983). Identification of elderly people with hearing problems. Asha..

[4] Lichtenstein MJ, Bess FH, Logan SA (1988). Diagnostic performance of the hearing handicap inventory for the elderly (screening version) against differing definitions of hearing loss. Ear Hear..

[5] Weinstein BE (1991). The Quantification of Hearing Aid Benefit in the Elderly. The Role of Self-assessment Measure. ACTA Otolaryngol..

[6] Corrêa GF, Russo ICP (1999). Autopercepção do handicap em deficientes auditivos adultos e idosos. Rev Cefac..

[7] Sindhusake D, Mitchell P, Smith W, Golding M, Newall P, Hartley D (2001). Validation of self-reported hearing loss. The Blue Mountains Hearing Study. Int J Epidemiol..

[8] Gates GA, Murphy M, Rees TS, Fraher A (2003). Screening for Handicapping hearing loss in the elderly. Fam Pract..

[9] Pinzan-Faria VM, Iorio MCM (2004). Sensibilidade auditiva e autopercepção de handicap: um estudo em idosos. Disturb Comum..

[10] Yueh B, Collins MP, Souza PE, Heagerty PJ, Liu CF, Boyko EJ (2007). Screening for Auditory Impairment - Which Hearing Assessment Test (SAI - WHAT): RCT design and baseline characteristics. Contemp Clin Trials..

[11] Russo ICP, Santos TMM (1994). Audiologia Infantil.

[12] Silman S, Silverman CA, Silman S, Silverman CA (1997). Auditory Diagnosis-Principles and applications.

[13] Davis H (1970). Hearing and Deafness.

[14] Russo IP, Russo IP (1999). Intervenção Fonoaudiológica na Terceira Idade.

[15] Wieselberg MB (1997). A Auto-avaliação do handicap em idosos portadores de deficiência auditiva: o uso do H.H.I.E. [mestrado].

[16] Wiley TL, Cruickshankst KJ, Nondahlt DM, Tweeds TS (2000). Self-Reported Hearing Handicap and Audiometric Measures in Older Adults. J Am Acad Audiol..

